# Cloning and characterization of microRNAs from wheat (*Triticum aestivum *L.)

**DOI:** 10.1186/gb-2007-8-6-r96

**Published:** 2007-06-01

**Authors:** Yingyin Yao, Ganggang Guo, Zhongfu Ni, Ramanjulu Sunkar, Jinkun Du, Jian-Kang Zhu, Qixin Sun

**Affiliations:** 1 Key Laboratory of Crop Heterosis and Utilization (MOE) and State Key Laboratory for Agrobiotechnology, Key Laboratory of Crop Genomics and Genetic Improvement (MOA), Beijing Key Laboratory of Crop Genetic Improvement, China Agricultural University, Beijing, 100094, China; 2 National Plant Gene Research Centre (Beijing), Beijing 100094, China; 3 Department of Biochemistry and Molecular Biology, Oklahoma State University, Stillwater, OK74078, USA; 4 Department of Botany and Plant Sciences, University of California, Riverside, CA 92521, USA

## Abstract

**Background:**

MicroRNAs (miRNAs) are a class of small, non-coding regulatory RNAs that regulate gene expression by guiding target mRNA cleavage or translational inhibition. So far, identification of miRNAs has been limited to a few model plant species, such as *Arabidopsis*, rice and *Populus*, whose genomes have been sequenced. Wheat is one of the most important cereal crops worldwide. To date, only a few conserved miRNAs have been predicted in wheat and the computational identification of wheat miRNAs requires the genome sequence, which is unknown.

**Results:**

To identify novel as well as conserved miRNAs in wheat (*Triticum aestivum *L.), we constructed a small RNA library. High throughput sequencing of the library and subsequent analysis revealed the identification of 58 miRNAs, comprising 43 miRNA families. Of these, 35 miRNAs belong to 20 conserved miRNA families. The remaining 23 miRNAs are novel and form 23 miRNA families in wheat; more importantly, 4 of these new miRNAs (miR506, miR510, miR514 and miR516) appear to be monocot-specific. Northern blot analysis indicated that some of the new miRNAs are preferentially expressed in certain tissues. Based on sequence homology, we predicted 46 potential targets. Thus, we have identified a large number of monocot-specific and wheat-specific miRNAs. These results indicate that both conserved and wheat-specific miRNAs play important roles in wheat growth and development, stress responses and other physiological processes.

**Conclusion:**

This study led to the discovery of 58 wheat miRNAs comprising 43 miRNA families; 20 of these families are conserved and 23 are novel in wheat. It provides a first large scale cloning and characterization of wheat miRNAs and their predicted targets.

## Abstract 2

A small RNA library was used to identify 58 miRNAs from 43 miRNA families from wheat (*Triticum aestivum *L.), and 46 potential targets were predicted.

## Background

MicroRNAs (miRNAs) are single-stranded noncoding RNAs ranging in size from approximately 20-22 nucleotides (nt). These are evolutionarily conserved across species boundaries and are capable of regulating the expression of protein-coding genes in eukaryotes [1]. miRNAs were first identified in *Caenorhabditis elegans *through genetic screens for aberrant development [2,3] and were later found in a number of multi-cellular eukaryotes using experimental and computational approaches [4]. In plants, most miRNAs were found through experimental approaches [5-12], although computational approaches were successful in identifying conserved miRNAs [13-16]. Most miRNA genes in plants exist as independent transcriptional units, have the canonical TATA box motif upstream of the transcriptional start site and are transcribed by RNA polymerase II into long primary transcripts (pri-miRNA) with 5' caps and 3' poly (A) tails [4,17-20]. miRNAs are generated from longer hairpin precursors by the ribonuclease III-like enzyme Dicer (DCL1) and possibly exported to the cytoplasm [4,21]. The miRNA:miRNA* duplex is unwound and the miRNA, but not miRNA*, is preferentially incorporated in the RNA-induced silencing complex (RISC) [4], functioning as a guide RNA to direct the post-transcriptional repression of mRNA targets, while the miRNA* is degraded [22,23].

Thus far, 4,361 miRNAs have been discovered from various organisms (miRNA Registry, Release 9.0, October 2006) [24]. A total of 863 miRNAs from plants were deposited in the current edition of miRNA registry. These miRNAs include 131 from *Arabidopsis*, 242 from rice, 215 from *Populus*, 96 from maize, 72 from *Sorghum*, 39 from *Physcomitrella*, 30 from *Medicago truncatula*, 22 from soybean, and 16 from sugarcane. To date, wheat miRNAs have not been deposited in the miRNA registry. Only recently, Zhang *et al*. [25] predicted 16 miRNAs in wheat based on sequence homology with the available expressed sequence tag (EST) sequences.

miRNA identification relies largely on two approaches: cloning and sequencing of small RNA libraries, that is, an experimental approach [11,12,26]; and computational prediction of conserved miRNAs [25]. In plants, experimental approaches led to the identification of not only conserved miRNAs but also several plant species-specific miRNAs in *Arabidopsis*, rice, *Populus *and *Physcometrella *[10,11]. Many miRNA families are evolutionarily conserved across all major lineages of plants, including mosses, gymnosperms, monocots and dicots; for example, *AthmiR166*, *miR159 *and *miR390 *are conserved in all lineages of land plants, including bryophytes, lycopods, ferns and monocots and dicots [26-28]. This conservation makes it possible to identify homologs of known miRNAs in other species [25,29]. Several computational programs such as MIRscan [30,31] and MiRAlign [32] have been developed for identification of known miRNA homologs from organisms whose genome sequences are available. Using this approach, many conserved miRNAs in plants and animals have been successfully predicted [4,13-15,33]. The experimental approach remains the best choice for identification of miRNAs in organisms whose genomes have not been sequenced.

Identification of small RNAs from *Arabidopsis*, rice, *Populus *and *Physcometrella *revealed a wealth of new information on small RNAs and their possible involvement in development, genome maintenance and integrity, and diverse physiological processes [34]. Our current knowledge about the regulatory roles of miRNAs and their targets point to fundamental functions in various aspects of plant development, including auxin signaling, meristem boundary formation and organ separation, leaf development and polarity, lateral root formation, transition from juvenile-to-adult vegetative phase and from vegetative-to-flowering phase, floral organ identity and reproduction [1,34]. In addition to their roles in development, the plant miRNAs have been shown to play important roles in response to nutrient deprivation, and biotic and abiotic stresses [10,14,35-38].

Wheat is the most widely grown crop, occupying 17% of all cultivated land and providing approximately 55% of the worlds carbohydrates [39], and is, therefore, of great economic importance. Thus far, EST database searches have predicted 16 miRNAs belonging to 9 conserved miRNA families in wheat [25], but their processing into mature miRNAs and their tissue distribution is unknown. In this study, using high throughput sequencing of a wheat small RNA library, we identified 58 miRNAs belonging to 43 miRNA families. These results validate 20 conserved miRNA families. Most importantly, four monocot-specific miRNA families were identified, in addition to a large number of wheat-specific miRNAs. Thus, the present study represents the first large scale identification of wheat miRNAs using experimental approaches. We also predicted 46 genes as potential targets for these wheat miRNAs. Predicted target genes include not only transcription factors implicated in development but also other genes involved in a broad range of physiological processes.

## Results

**Figure 1:**
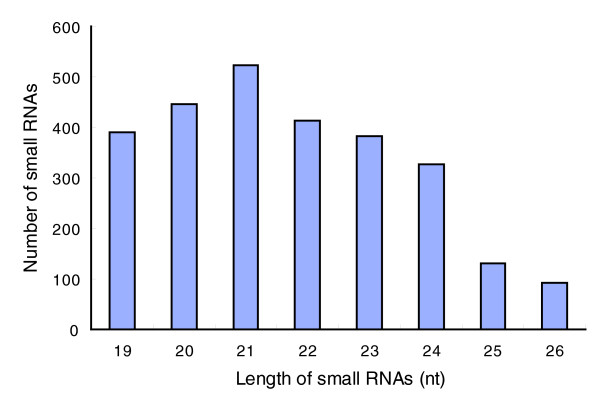
The size distribution of small RNAs.

In order to identify novel as well as conserved miRNAs in wheat, we generated one small RNA library ranging in size from 18-26 nt using pooled RNA isolated from leaves, roots and spikes. Pyrosequencing of the wheat small RNA library was performed at 454 Life Sciences™, and generated a total of 262,955 sequences. Analysis of these sequences resulted in identification of 25,453 unique sequences ranging in size from 18-26 nt in length. The remaining sequences were of low quality, had inserts smaller than 18 nt, representing degraded RNA, or were without inserts, and were excluded from further analysis. The majority of the small RNAs are 20-24 nt in size, which is the typical size range for Dicer-derived products and the 21 nt size class is predominant (Figure 1).

### Identification of new monocot-specific and wheat specific-microRNAs

One of the important features that distinguish miRNAs from other endogenous small RNAs is the ability of the miRNA surrounding sequences to adopt a hairpin structure [40]. Since the wheat genome is largely unknown, we have to rely on wheat EST sequences to predict hairpin structures on the basis of miRNA surrounding sequences. To identify atypical and new miRNAs in wheat or wheat-specific miRNAs, we adopted the following strategy. In the first step, we searched the EST databases that perfectly match the small RNA sequences. In the second step, these ESTs were searched against the Rfam database to remove non-coding RNAs such as rRNA, tRNA and so on. In the third step, the remaining ESTs were, in turn, used to search against a protein database to remove the degradation products from protein-coding sequences. And in the fourth step, the remaining EST sequences were used in predicting the fold-back structures and classified as new microRNAs (Table 1; Additional data file 1) or endogenous small RNAs (data not shown).

**Table 1:** Novel wheat miRNAs identified by direct cloning

Name	Sequence	Length (nt)	EST no.*	Unigene	EST length	Precursor length	Start, end	Energy kcal mol^-1^	Expression
TamiR501	UAGUACCGGUUCGUGGCACGAACC	24	CA718024	Ta.23206	168	83	20, 102	-67.20	Not detected
			CD878657	Ta.34663	551	151	92, 242	-82.40	
TamiR502	CACUACAUUAUGGAAUGGAGGGA	23	CA670378	Ta.2228	550	245	216, 460	-94.10	Northern blot
TamiR503	UGGCACGGCGUGAUGCUGAGUCAG	24	BG262612	Ta.14534	474	70	340, 409	-36.3	Not tested
TamiR504	ACAUUCUUAUAUUAUGAGACGGAG	24	CA739366	Ta.28672	427	87	14, 100	-68.6	RT-PCR
TamiR505	AGUAGUGAUCUAAACGCUCUUA	22	BJ323011	Ta.38265	690	87	248, 334	-63.8	RT-PCR
			BJ263967	Ta.2752	464	115	78, 192	-49.9	
			CA694693	Ta.12686	491	88	92, 180	-41.4	
TamiR506	UAGAUACAUCCGUAUCUAGA	20	CK214157	Ta.32635	1,048	126	140, 265	-89.3	RT-PCR
			BE430261	Ta.38727	558	128	292, 420	-69.3	
			BJ267812	Ta.14358	179	129	10, 138	-80.4	
TamiR507	UCCGUGAGACCUGGUCUCAUAGA	23	CK217185	Ta.30511	1,047	181	550, 730	-82.4	Northern blot
			AY747601	-	-	218	1, 218	-154.3	
TamiR508	GCAGGACGUGAAGAGCGAGUCC	22	BE417418	Ta.23807	310	115	155, 269	-52.70	RT-PCR
TamiR509	AACCAACGAGACCAACUGCGGCGG	24	CA635339	Ta.2228	583	179	190, 368	-87.8	Northern blot
TamiR510	UCCACUAUGGACUACAUACGGAG	23	AJ603161	Ta.639	429	163	95, 257	-70.1	Not detected
TamiR511	UCCUUCCGUUCGGAAUUAC	19	BE405744	Ta.30840	545	116	260, 375	-42.3	Not tested
TamiR512	UACUACUCCCUCCGUCCGAAA	21	BJ320481	Ta.7082	439	133	90, 222	-86.9	Northern blot
TamiR513	CAGCGAGCCAGCGGAGACCGGCAG	24	BJ260462	Ta.6068	572	298	220, 517	-138.0	Northern blot
TamiR514	CCUCCGUCUCGUAAUGUAAGACG	23	CA676805	Ta.14883	625	113	20, 132	-51.2	Northern blot
TamiR515	UAGUACCGGUUCGUGGCUAACC	22	CA686406	Ta.22812	544	67	333, 399	-43.9	Northern blot
TamiR516	AUAGCAAGGAUUGACAGACUG	21	BJ215780	Ta.25530	608	551	50, 600	-172.9	Not tested
TamiR517	CAUAUACUCCCUCCGUCCGAAA	22	BJ276129	Ta.33730	281	145	50, 194	-76.9	Not tested
TamiR518	CAACAACAACAAGAAGAAGAAGAU	24	BE442798	Ta.8114	588	379	91, 469	-145.1	Not tested
TamiR519	CUGCGACAAGUAAUUCCGAACGGA	24	CA698039	Ta.28713	429	109	72, 180	-60.3	Not tested
			DR092358	Ta.41690	250	109	100, 208	-64.0	
TamiR520	UUGUCGCAGGUAUGGAUGUAUCUA	24	BE591362	Ta.2140	463	106	145, 250	-68.8	Not tested
TamiR521	UAGUACAAAGUUGAGUCAUC	20	BJ237878	Ta.3199	685	123	109, 231	-70.0	Not tested
			BQ172311	Ta.12786	474	89	62, 150	-60.9	
TamiR522	GCUUAGAUGUGACAUCCUUAAAA	23	DR733919	Ta.12590	930	147	300, 446	-32.0	Not tested
TamiR523	AGAGUAACAUACACUAGUAACA	22	BQ903908	Ta.27907	636	207	423, 629	-67.4	Not tested
TamiR524	CAUUAUGGAACGGAAGGAG	19	BJ241591	Ta.9978	328	90	141, 230	-46.5	Not tested

* ESTs belonging to same unigene cluster were not included in this table.

Our analysis revealed that 4,744 sequences matched at least 1 wheat EST and these were analyzed further. As determined by BLASTn and BLASTx searches against the Rfam database and protein database, 2,039 sequences represented the fragments of abundant non-coding RNAs (rRNA, tRNA, small nuclear RNA and small nucleolar RNA). The remaining 2,705 sequences constitute miRNAs (Tables 1 and 2) and endogenous small interfering RNAs (siRNAs; data not shown). Our search for new miRNAs revealed that 23 sequences that perfectly matched ESTs were able to adopt hairpin structures and these comprise 23 new miRNA families (Table 1). The lengths of these newly identified miRNAs vary from 19 to 24 nt, and 10 of the 23 novel miRNAs begin with a 5' uridine, which is a characteristic feature of miRNAs.

**Table 2:** Conserved wheat miRNA families homologous to known miRNAs from other plant species

miRNA family	Name	Sequence(5'-3')*	Length (nt)	Pri-miRNA EST no.	Conserved in other plants^†^			
					
					Rice	*Arabidopsis*	Maize	Sorghum
156/157	TaMIR156a	UGACAGAAGAGAGUGAGCAC	20	Not found	++	++	++	++
	TaMIR156k	UUGACAGAAGAGAGUGAGCA	20		+	+	+	+
	Ta MIR156m	UGACAGAAGAGAGUGAGCCU	20		+	+	+	+
159	TaMIR159a	UUUGGAUUGAAGGGAGCUCUG	21	CA731881	++	+	++	++
	TaMIR159b	UUUGGAUUGAAGGGAGCUCUU	21	CA484819 CA682604	+	++	+	+
160	TaMIR160	UGCCUGGCUCCCUGUAUGCCA	21	CJ641547	++	++	++	++
164	TaMIR164a	UGGAGAAGCAGGGUACGUGCA	21	CA704421	++	++	++	++
165	TaMIR165	UCGGACCAGGCUUCAUCCCC	20	Not found		+		
166	TaMIR166b	UCGGACCAGGCUUCAUUCCC	20	Not found	++	++	++	++
	TaMIR166g	UCGGACCAGGCUUCAAUCCC	20		++	++	++	++
167	TaMIR167a	UGAAGCUGCCAGCAUGAUCUA	21	CK209908	++	++	++	++
	TaMIR167g	UGAAGCUGCCAGCAUGAUCUG	21	CK209889	++	++	++	++
	TaMIR167m	UGAAGCUGCCAGCAUGAUCUGA	22		+	+	+	+
168	TaMIR168a	UCGCUUGGUGCAGAUCGGGAC	21	Not found	++	+	++	++
	TaMIR168b	UCGCUUGGUGCAGAUCGGGAU	21		+	+	+	+
169	TaMIR169a	CAGCCAAGGAUGACUUGCCGA	21	BJ225371	++	++	++	++
	TaMIR169b	CAGCCAAGGAUGACUUGCCGG	21		++	++	++	++
	TaMIR169n	ACAGCCAAGGAUGACUUGCCG	21		+	+	+	+
	TaMIR169m	UAGCCAAGGAUGACUUGCCUG	21		++	++	++	++
	TaMIR169o	UAGCCAAGGAUGACUUGCCUA	21		++	++	++	++
170/171	TaMIR171a	UGAUUGAGCCGUGCCAAUAUC	21	CD910903	++	++	++	++
	TaMIR171b	UUGAGCCGUGCCAAUAUCACG	21		+	++	+	+
	TaMIR171h	GUGAGCCGAACCAAUAUCACU	21		++	+	++	++
172	TaMIR172a	AGAAUCUUGAUGAUGCUGCAU	21	Not found	++	++	++	++
	TaMIR172n	GAAUCUUGAUGAUGCUGCAU	20		+	+	+	+
	TaMIR172c	UGAAUCUUGAUGAUGCUGCAU	21		+	+	+	+
319	TaMIR319a	UUGGACUGAAGGGUGCUCCC	20	Not found	++	+	++	++
	TaMIR319d	UUUGGAUUGAAGGGAGCUCU	20	Not found				
390	TaMIR390	AAGCUCAGGAGGGAUAGCGCC	21	Not found	++	++		
393	TaMIR393	UCCAAAGGGAUCGCAUUGAUC	21	Not found	++	++	++	++
396	TaMIR396a	UUCCACAGCUUUCUUGAACUG	21	Not found	++	++	++	++
397	TaMIR397	UUGAGUGCAGCGUUGAUGAA	20	Not found	+	+		
399	TaMIR399	UGCCAAAGGAGAAUUGCCC	19	CJ666653	+	+	+	+
408	TaMIR408	CUGCACUGCCUCUUCCCUGGC	22	BE419354	++	++	++	
444	TaMIR444	UUGCUGCCUCAAGCUUGCUGC	21	CK200584	++			
				CA596074				
				BE405735				
479	TaMIR479	AGUGAUAUUGGUCCGGCUCAUU	22	Not found				

The sequences given in this table represent the longest miRNA sequences identified by cloning and 454 sequencing. *The underlined nucleotides represent the non-conserved nucleotides among wheat and other plant species. ^†^The plus symbols indicate: ++, miRNA sequences of wheat were exactly identical to those in other species; +, miRNA sequences of wheat were conserved in other species but have variations in some nucleotide positions.

Our newly identified wheat miRNA precursors have negative folding free energies (from -32 to -172.9 kcal mol^-1 ^with an average of about -72.4 kcal mol^-1^) according to MFOLD, which is similar to the free energy values of other plant miRNA precursors (-71.0 kcal mol^-1 ^in rice and -59.5 kcal mol^-1 ^in *Arabidopsis*). These values are much lower than folding free energies of tRNA (-27.5 kcal mol^-1^) or rRNA (-33 kcal mol^-1^) [41]. The predicted hairpin structures for the precursors of these miRNAs require 67-551 nt, with a majority of the identified miRNA precursors (74.2%) requiring 67-150 nt, similar to what has been observed in *Arabidopsis *and rice [42]. The predicted secondary structures indicate that at least 16 nucleotides are engaged in Watson-Crick or G/U base pairings between the mature miRNA and the miRNA* in the hairpin structure [43]. We also analyzed the secondary structure of the miRNAs and miRNAs*. Based on the method proposed by Dezulian *et al*. [16], we scored the strength of the bond at each position of the miRNA and miRNA*. Different values were given to the different base pairs: GC was given a score of 3; AU a score of 2; GU a score of 1; and unpaired nucleotides a score of 0. This analysis indicated that the average strength score of the 5' nucleotide of 23 novel miRNAs is 1.6, whereas the average strength score of the 5' nucleotide of the corresponding miRNAs* is 2.3. These scores are highly similar to those in other plant species (1.6 for miRNA and 2.4 for miRNA*) [16]. These features of the novel wheat miRNAs are consistent with previous reports in animals and plants where the first nucleotide of the miRNA is more likely to be unpaired than the first nucleotide of the miRNA*. Thus, 23 of these small RNAs satisfied the criteria to be categorized as novel miRNAs in wheat.

To determine whether these novel miRNAs are conserved among other plant species, we searched the nucleotide databases for homologs. This analysis indicated that four miRNAs, TamiR506, TamiR510, TamiR514 and TamiR516, are conserved in other monocots, such as rice, barley and *Festuca arundanacea*. Hairpin structures can be predicted for these miRNAs from rice, barley and *Festuca arundanacea *using miRNA surrounding sequences obtained from ESTs. These findings indicate that these four miRNAs are conserved in monocots but not in *Arabidopsis *or *Populus*, suggesting that these are monocot-specific miRNAs.

**Figure 2:**
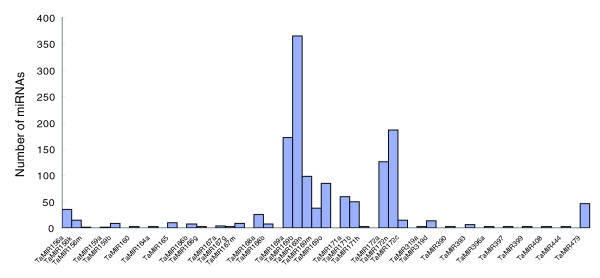
The frequency of conserved miRNAs present in the sequenced small RNA library.

Interestingly, we found that one miRNA, TamiR507, mapped to the wheat genome by searching the NCBI database. This locus resides in the promoter region of the gene VRN-A1 (AY747601). The genomic sequence has high (73%) nucleotide similarity in the stem-loop region with EST CK217185, the precursor of TamiR507. Both the EST and genomic sequence can form a hairpin structure, and the miRNA was detected on small RNA gel blots as a discrete band (Figure 2), suggesting that it is not a degradation product. The existence of miRNA loci in promoter regions was hitherto unknown, and most miRNAs map to intergenic regions and only a few to introns or exons [11].

### Identification of conserved miRNAs in wheat

To identify the conserved miRNA homologs in wheat, we analyzed the small RNA library for the presence of known miRNAs. We used BLASTN with an E-value cutoff of 10 for the similarity search against the central miRNA Registry Database [44]. Using this search, a total of 35 miRNAs belonging to 20 conserved miRNA families were identified (Table 2). These include miRNA156/157, miR159, miR160, miR164, miR165/166, miR167, miR168, miR169, miR170/171, miR172, miR319, miR390, miR393, miR396, miR397, miR399 and miR408, which are conserved in diverse plant species (Table 2). In addition, we also found miR444 in a wheat small RNA library; miR444 is a monocot-specific miRNA [45]. Several of the conserved miRNA precursors were found in EST sequences [16,42,45], although miRNA precursors are relatively under-represented in ESTs, possibly because miRNA processing is rapid and miRNA precursors were rarely detected using Northern analysis in plants. Nevertheless, in the absence of genome sequence information on target plant species, an EST database could be used as a source for miRNA precursor sequences. miRNA sequence homology searches against ESTs were performed to search for the conserved miRNA precursors. This analysis revealed perfect matching of nine miRNA families, miR159, miR160, miR164, miR167, miR169, miR170, miR399, miR408 and miR444, to 14 ESTs. All these EST sequences, which are also miRNA precursors, can adopt hairpin structures resembling previously known miRNA fold-back structures (Additional data file 1). Some of these miRNA families (for example, miR319, miR390, and miR165/166) are conserved deeply, including in lower plants such as *Physcometrella *[26-28].

The number of times each miRNA is represented in the small RNA library could serve as an index for the estimation of the relative abundance of miRNAs. The large number of miRNA sequences generated in this study would allow us to determine the relative abundance of miRNAs in wheat. The frequencies of the miRNA families varied from 2 (miR390, miR396, miR397, miR399) to 757 (miR169), indicating that expression varies highly among the different miRNA families in wheat (Figure 2).

MiRNAs can be grouped into families based on sequence similarity. Sequence analysis revealed nine conserved miRNA families represented by more than one member in our library. MiR169 was represented by five members, miR156, miR165/166, miR167, miR170/171 and miR172 were represented by three members each, and miR159, miR319 and miR168 were represented by two members each in the library. Furthermore, our analysis revealed that the library included all known members of several miRNA families: miR156, miR159, miR167, miR169, miR168, miR171 and miR172. Using Northern blot analysis, it is almost impossible to differentiate between the expression levels of miRNA family members. High throughput sequencing of the small RNA libraries allowed us to identify the expression levels of each member within a family. Sequence analysis indicated that the relative abundance of certain members within the miRNA families varied greatly (Figure 2). For instance, miR169b and miR169a appeared 365 and 171 times, respectively, whereas the other three members (miR169m, miR169n and miR169o) appeared between 38 and 98 times. Similarly, miR172n and miR172a appeared 186 and 126 times, respectively, whereas miR172c appeared only 14 times. MiR168a appeared 25 times, whereas miR168b was found 7 times in the library. miRNA members of the miR156 family also showed variable expression. These results indicate that certain members within a miRNA family show preferential expression, which could be attributed to high level tissue-specific expression of these members.

### Expression patterns of conserved and newly identified microRNAs in wheat

Knowledge about the expression patterns of miRNAs might provide clues about their functions. To get an insight into possible stage- or tissue/organ-dependent roles of miRNAs in wheat, we examined the expression patterns of miRNAs in different tissues, including roots and leaves of seedlings, nodal regions, spikes, internodes just below the spike, and flag leaf of the booting stage.

**Figure 3:**
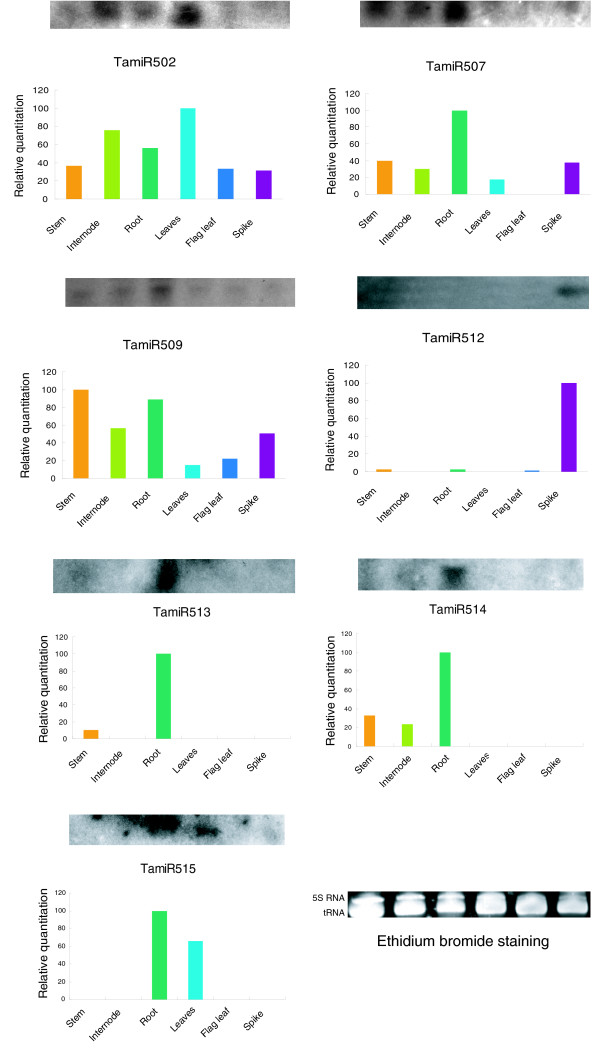
Expression patterns of novel miRNAs in wheat. RNA gel blots of low molecular weight RNA from different tissues, including stems, internodes below spikes, leaves, flag leaves, roots and spikes, were probed with labeled oligonucleotides. The tRNA and 5S RNA bands were visualized by ethidium bromide staining of polyacrylamide gels and served as loading controls.

To confirm the expression of novel miRNAs in wheat tissues, we performed Northern analyses in different tissues/organs. Out of 13 novel miRNAs tested, 7 could be detected, whereas the remaining 6 could not be detected using small RNA gel blot analysis. However, using RT-PCR, we confirmed the expression of four of the novel miRNA precursors, indicating that their expression is relatively low. Taken together, the expression of 11 novel wheat miRNAs was detectable using RNA gel blot or PCR analyses. The expression of miR502, miR507, miR509, miR512, miR513, miR514 and miR515 was detectable by RNA gel blot analysis (Figure 3). MiR502 seemed to be strongly expressed in internodes, roots and leaves but was barely detected in stems and spikes. MiR507 and miR509 had similar expression patterns: they were expressed abundantly in roots, moderately in stems and internodes and weakly in leaves, spikes and flag leaves. MiR512 showed tissue-specific expression and was detected only in spikes. MiR513 and miR514 also exhibited tissue-specific expression, being expressed in roots only. MiR515 expression appeared to be restricted to roots and leaves (Figure 3).

**Figure 4:**
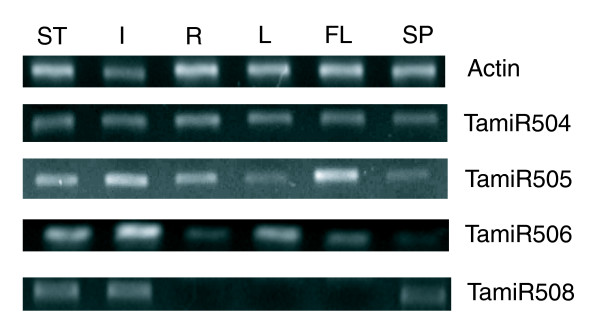
Semi-quantitative RT-PCR analyses of novel miRNAs in wheat. Relative expression of miRNAs in stems, internodes below spikes, leaves, flag leaves, roots and spikes was analyzed by semi-quantitative RT-PCR. A wheat actin gene was selected to normalize the amount of templates added in the PCR reactions. ST, stems; I, internodes below spikes; R, roots; L, leaves; FL, flag leaves; SP, spikes.

The expression of four wheat miRNAs (miR504, miR505, miR506 and miR508) was validated by semi-quantitative RT-PCR, as these could not be detected using Northern blot analysis (Figure 4). MiR505 and miR506 had low expression levels in spikes, and miR508 was found to be uniformly expressed in stems, internodes and spikes but could not be detected in leaves and roots. MiR504 showed ubiquitous expression in all the tissues examined (Figure 4).

**Figure 5:**
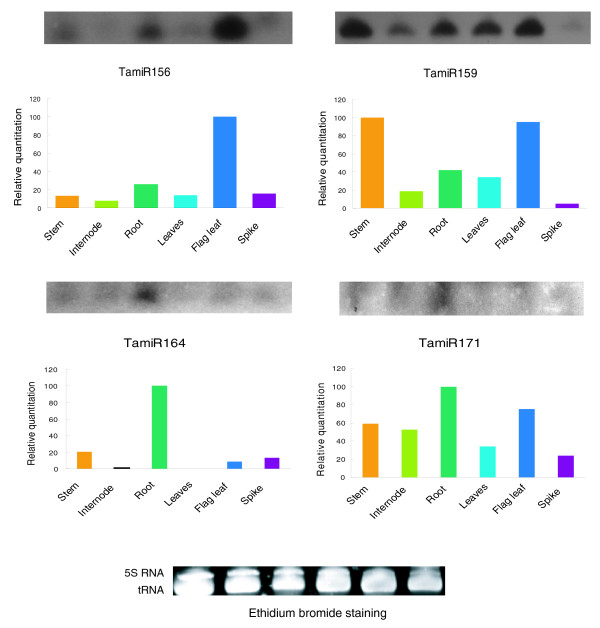
Expression patterns of conserved miRNAs in wheat. RNA gel blots of low molecular weight RNA from different tissues, including stems, internodes below spikes, leaves, flag leaves, roots and spikes, were probed with labeled oligonucleotides. The tRNA and 5S RNA bands were visualized by ethidium bromide staining of polyacrylamide gels and served as loading controls.

The expression patterns of miR156, miR159, miR164, and miR171, which are conserved miRNAs, were examined by RNA gel blot analysis (Figure 5). Expression of miR156 was higher in roots and flag leaves, but lower in other tissues tested, especially in spikes. MiR159 was found to be strongly expressed in all tissues examined except in spikes, in which the expression levels were low. MiR164 showed moderate expression in roots and was barely detectable in other tissues. MiR171 showed ubiquitous expression in all tissues, although the expression in roots was relatively higher (Figure 5). These observations suggest that these miRNAs display differential tissue-specific expression patterns.

### Target predictions for wheat miRNAs

**Figure 6:**
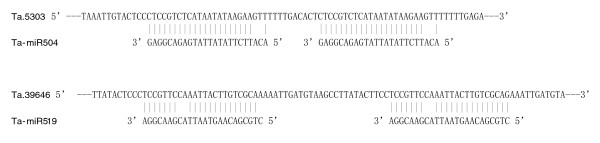
Wheat unigenes Ta.5303 and Ta.39646, the predicted targets of miRNA 504 and miRNA 519, respectively, were both found to have two complementary sites.

It has been reported that most target mRNAs of miRNAs in plants have one miRNA-complementary site located in coding regions and occasionally in the 3' untranslated regions (UTRs) or 5' UTRs [10,11,14,33,46], and that plant miRNAs exhibit perfect or near perfect complementarity with their target mRNAs [47]. We adopted a set of rules proposed in earlier reports for predicting miRNA targets [11,48]. These criteria include allowing one mismatch in the region complementary to nucleotide positions 2 to 12 of the miRNA, but not at position 10/11, which is a predicted cleavage site, and three additional mismatches between positions 12 and 22 but with no more than two continuous mismatches. To identify potential targets for wheat miRNAs, we searched for antisense hits in wheat EST and Unigene sequences. In plants, the miRNA target sites were found predominantly in the coding regions [10,11,15]. Consistent with these findings, 29 of our predicted target genes have target sites in the coding region; 15 target genes have miRNA complementary sites in 3' UTRs whereas 2 target genes were found to have miRNA target sites in 5' UTRs. Interestingly, wheat unigenes Ta.5303 and Ta.39646, which are likely to be targeted by miR504 and miR519, were found to have two complementary sites. Both target sites were very closely spaced and separated by 10 nucleotides in Ta.5303 and are perfectly complimentary to miR504 (Figure 6). In Ta.39646, the two sites are also closely spaced and separated by 25 nucleotides (Figure 6).

Regulatory targets can be more confidently predicted for conserved miRNAs since complementary sites are also conserved across different species [10,14,45]. In this study, our search predicted 30 unigenes as putative targets for 20 conserved miRNAs (Additional data file 2). As expected, these target genes were similar or related to the previously validated plant miRNA targets in *Arabidopsis*, rice and *Populus *[10,13-15,33,45,46]. Twelve conserved miRNA families (miR156/157, miR159/319, miR160, miR164, miR165/166, miR167, miR169, miR170/171, miR172 and miR444) have been predicted to target 24 transcription factors, including squamosa promoter binding proteins, MYB, NAC1, homeodomain-leucine zipper protein, auxin response factor, CCAAT-binding protein, scarecrow-like protein, APETELA2 protein and MADS box protein (Additional data file 2). MiR393 is likely to target Ta.23215, which encodes transport inhibitor response (TIR)1, and three other related members (Ta.1725, Ta.20960 and Ta.30891). MiR408 could target blue copper proteins (plantacyanins) and wheat miR168 targets argonaute, which is encoded by Ta.34670 and Ta. 2949 (Additional data file 2). TIR1, plantacyanin and argonaute have been validated as genuine targets of miR393, miR408 and miR168 in *Arabidopsis*, rice and *Populus *[10,11,13,28,46,49].

We also predicted 16 unigenes to be putative targets for 12 newly identified miRNAs (Additional data file 2). These target genes belong to several gene families predicted to play roles in a broad range of physiological processes. Of these 16 targets, 3 appear to be involved in the defense response. These include aspartic-type endopeptidase/pepsin A, putative UVB-resistance protein, and early light-inducible protein (ELIP). Other putative targets include transcription elongation factor 1, translation initiation factor 4B, ferric reductase, binding protein, and expansin like protein A. Interestingly, miR506 is predicted to target AB182944, which encodes a knox1b homeobox protein, a transcription factor. We also predicted CRT/DRE binding factor to be a putative target of miR507. These two genes have not been previously predicted as putative miRNA targets in plants. We also predicted six target genes with unknown functions as miRNA targets in wheat. These observations suggest that microRNA targeted genes in wheat play roles not only in development but also in diverse physiological processes.

We were unable to predict targets for 11 of the new miRNAs (miR501, miR503, miR508, miR510, miR511, miR515, miR516, miR517, miR518 miR520 and miR523) by applying the above rules, which could be due to the limited number of wheat EST sequences available in the databases.

## Discussion

The identification of entire sets of miRNAs and subsequently their targets will lay the foundation to unravel the complex miRNA-mediated regulatory networks controlling development and other physiological processes. Several computational studies have estimated that miRNA genes probably comprise 1% of the total protein-coding genes of organisms [30,31,50]. In humans and other primates, the amount of miRNA has gone beyond these estimations. It is also proposed that about 30% of all human genes may be regulated by miRNAs [30,31,50]. To date, 863 miRNA sequences have been identified from plant species. However, only nine conserved miRNA families were computationally predicted in wheat [25]. Experimental approaches in *Arabidopsis*, rice, *Popupus *and *Physcometrella *have been instrumental in finding miRNAs that, in addition to conserved miRNAs, are conserved only in closely related plant species or that are even plant species-specific [10-12,26]. In this study, using an experimental approach, we provide evidence for the existence of 20 conserved miRNA families as well as 23 novel miRNA families in wheat. Four of these new miRNAs were found to be conserved in other monocots such as rice, barley and *F. arundinacea*, suggesting that they are monocot-specific. However, we can not find homologs of the remaining 19 miRNAs in other plants, and these might represent wheat specific miRNAs. Several miRNAs are conserved, often over wide evolutionary distances. Up to now, miRNA identification in monocotyledonous plants using a cloning approach has been limited to rice and led to identification of few monocot-specific miRNAs [45]. In this study, by using another monocot, cloning led to the identification of four additional miRNAs that are specific to monocots. Future large scale experimental approaches in monocots are likely to identify additional monocot-specific miRNAs.

### Wheat miRNAs differ in their expression patterns compared to those in *Arabidopsis *and rice

Knowledge about the expression of miRNAs might provide clues about where these miRNAs function. Previous reports have indicated that several *Arabidopsis*, rice and *Populus *miRNAs are expressed ubiquitously while the expression of many others is regulated by development and show preferential accumulation in certain tissues [5,6,8,10,14], and some others are regulated in response to stress [10,14,35-38]. The expression analysis of TamiR156 revealed a similar tissue-specific expression pattern to that in *Arabidopsis*. TamiR156 showed higher expression levels in stem, roots and flag leaves, but lower levels in other tissues tested, especially in spikes. In *Arabidopsis*, miR156 was strongly expressed during seedling development and showed weak expression in mature tissues [28]. Rice miR156 showed similar expression profile to those found in *Arabidopsis *and wheat [51]. However, some other conserved miRNAs showed markedly different expression patterns in wheat compared to *Arabidopsis *or rice. For example, TamiR159 seems to be strongly expressed in all tissues examined with the exception of spikes, where the expression levels seem to be low. In contrast, rice miRNA159 is highly expressed in floral organs [52]. TamiR164 showed high expression levels in roots but was barely detectable in other tissues. However, *Arabidopsis *miR164 displayed higher levels of expression in roots and inflorescences than in leaves [53,54]. TamiR171 showed ubiquitous expression in all tissues, although the expression in roots was relatively higher. However, this expression pattern differed markedly from that of its conserved *Arabidopsis *counterpart, which is highly expressed in flowers [6]. Similarly, the expression patterns of 11 *Populus *miRNAs that are conserved in *Arabidopsis *are not similar in both plant species [12]. These findings suggest that although miRNAs are conserved, their expression patterns can differ among different plant species.

### Predicted targets of wheat miRNAs might play roles in a broad range of biological functions

More recent studies have demonstrated that miRNAs in *Arabidopsis*, rice and other plant species target transcripts encoding proteins involved in diverse physiological processes [11-15,33], among which a set of miRNAs predominantly targeted transcription factors. In this study, we were able to predict 46 unigenes as putative miRNA targets in wheat, with one-third of the predicted targets of miRNAs being transcripts encoding transcription factors, including squamosa promoter binding protein, MYB, NAC, ARF, HD-Zip, Scarecrow like proteins and Apetala2. Other target genes include those encoding argonaute protein, TIR1, basic blue copper protein, aspartic-type endopeptidase/pepsin A, transcription elongation factor 1, ferric reductase, putative UVB-resistance protein, binding protein, ELIP, and expansin like protein A, suggesting that wheat miRNAs are involved in a broad range of physiological functions. Further analysis indicated that target genes of 12 conserved wheat miRNAs are also conserved among other plant species, implying that conserved miRNAs play conserved biological functions. Moreover, 16 targets, especially for non-conserved miRNAs, were distinct from *Arabidopsis *and rice genes, indicating that these targets may be involved in wheat specific processes. It will be an interesting area to identify the functions of these predicted target genes in wheat.

Most target mRNAs of plant miRNAs have only one single miRNA-complementary site located in coding regions and occasionally in the 3' or 5' UTRs [10,11,14,33,46]. Consistent with these reports, wheat miRNAs are predicted to target coding regions. Although 3' UTRs are predicted as target sites for plant miRNAs in only a few cases in the previous reports, of the 16 targets of novel wheat miRNAs reported in this study, 11 are within 3' UTRs, only 3 are in a coding region, and 2 are in a 5' UTR. This bias might reflect a mechanistic preference for translational repression. Depending on the degree of miRNA complementarity with target mRNA, it appears that perfectly base-paired miRNAs mediate cleavage, and the imperfectly base-paired miRNAs mediate translation repression [55]. We found that half of miRNAs targeting 3' UTRs were perfectly base-paired, and they might cleave the target mRNA to down-regulate its expression. Rice miR439 had been reported to have three complementary sites within a coding region of the target mRNA [11]. Future experiments will reveal whether these target genes are destined for degradation or translational repression.

## Conclusion

Cloning of small RNAs is a starting point to understand their number, diversity and possible roles in different organisms. Recent studies have clearly indicated the importance of small RNA cloning, particularly in the identification of non-conserved atypical miRNAs in diverse species, such as *Arabidopsis*, rice, *Populus *and *physcometrella *[6,8,10,12,20,26,45]. This study led to the discovery of 58 wheat miRNAs comprising 43 miRNA families, of which 20 and 23 belong to conserved and novel wheat miRNA families, respectively. Importantly, we have identified four monocot-specific miRNAs. We further show that some of the miRNAs are differentially expressed in a tissue- or developmental stage-dependent manner. This study provides a first large scale cloning and characterization of wheat miRNAs and their predicted targets, which serve as a foundation for future functional studies.

## Materials and methods

### Plant materials

Hexaploid wheat (*Triticum aestivum *L.) line 3338 was grown in a growth chamber at a relative humidity of 75% and 26/20°C day/night temperature with light intensity of 3000 lx. Leaves and roots from one-month-old seedlings, and spikes at booting stage were collected and used for generation of small RNA libraries. For expression analysis, seedling roots and leaves, nodal regions (stems at jointing stage), spikes, the internode below the spike, and flag leaves at booting stage were collected and used.

### Cloning of wheat miRNAs

Total RNA was isolated from the leaves, roots and spikes using the Trizol (Invitrogen, Carlsbad, CA, USA) according to the manufacturer's instructions, and then pooled. Cloning of the miRNAs was performed as described by Sunkar and Zhu [10]. Briefly, low molecular weight RNA was enriched by 0.5 M NaCl and 10% PEG8000 precipitation. About 100 μg of low molecular weight RNA was separated on a denaturing 15% polyacrylamide gel. RNA oligonucleotides labeled at positions 18 and 26 were used as size standards. The nucleotides from positions 18-26 were excised, and RNA was eluted overnight with 0.4 M NaCl at 4C. The RNA was dephosphorylated by alkaline phosphatase (New England Biolabs Inc., Beijing, China) and recovered by ethanol precipitation. The small RNAs were then ligated sequentially to 5' (5'-tactaatacgactcactAAA-3'; uppercase, RNA; lowercase, DNA) and 3' (5'-pUUUaaccgcatccttctcx-3'; uppercase, RNA; lowercase, DNA; p, phosphate; x, inverted deoxythymidine) RNA/DNA chimeric oligonucleotide adapters. Reverse transcription was preformed after ligation with adapters, followed by PCR amplification. The resulting PCR products were sequenced using 454 Life Sciences™ technology [56] as described [57].

### Data analysis

Automated base calling of the raw sequences and vector removal were performed with PHRED and CROSS MATCH programs [10,11]. All trimmed sequences between 19 and 26 bp in length were used to search the Rfam database [58] with BLASTN [59] to remove most non-siRNA and non-miRNA sequences. Putative origins for the remaining sequences were identified by BLASTN search against the wheat EST database from NCBI. The protein-coding EST sequences were removed and the remaining non-coding candidate wheat ESTs with perfect matches with small RNA sequences were used for fold back secondary structure prediction with the MFOLD program [9]. In the NCBI Unigene database, closely related wheat ESTs have been assembled in the Unigene cluster; therefore, the Unigene accessions were selected and recorded. Based on these analyses, putative miRNAs were then searched against the NCBI NT database to check whether these miRNAs exist in other species.

Target predictions were performed by searching the wheat EST database and NCBI NT database for miRNA complementary sequences, allowing up to three mismatches and with no gaps between the miRNA and target mRNA.

### RNA gel blot analysis

Low molecular weight RNA was isolated from leaves, roots, stems, spikes, internodes below spikes and flag leaves. Low molecular weight RNA (30 μg) was loaded per lane, resolved on a denaturing 15% polyacrylamide gel, and transferred electrophoretically to Hybond-N+ membranes (Amersham Biosciences, Buckinghamshire, UK). Membranes were UV cross-linked and baked for 2 hours at 80°C. DNA oligonucleotides complementary to miRNA sequences were end-labeled with γ-^32^P-ATP using T4 polynucleotide kinase (TaKaRa, Dalian, China). Membranes were prehybridized for more than 8 hours and hybridized overnight using Church buffer at 38°C. Blots were washed three times (two times with 2 × SSC + 1% SDS and one time with 1 × SSC + 0.5% SDS) at 50°C. The membranes were briefly air dried and then exposed to X-ray films for autography at -80°C. Images were acquired by scanning the films with a FluorChem™ (Alpha Innotech, San Leandro, CA, USA). Signal intensities of spots were analyzed using FluorChem™ 5500 software.

### Semi-quantitative RT-PCR validation of MIRNA expression

Total RNA was isolated from leaves, roots, stems, spikes, internodes below spikes and flag leaves by using Trizol (Invitrogen) according to the manufacturer's instructions and treated with RNase-free DNase I (Promega, Madison, WI, USA). Total RNA (2 μg) from each sample was used for first-strand cDNA synthesis in 20 μl reactions containing 50 mM Tris-HCl (pH 8.3), 75 mM KCl, 3 mM MgCl_2_, 10 mM DTT, 50 μM dNTPs, 200 U M-MLV reverse transcriptase (Promega) and 50 pmol oligonucleotides T15. Reverse transcription was performed at 37°C for 60 minutes with a final denaturation at 95°C for 5 minutes. Gene-specific RT-PCR primers for four miRNA precursors were designed according to the EST sequences.

Three RT-PCR replications were conducted using independently isolated total RNAs with the following thermal cycling parameters: 94°C for 30 s, 57°C for 30 s, and 72°C for 30 s. A 350 bp β-actin gene fragment was amplified as a positive control using the primer pair 5'-CAGCAACTGGGATGATATGG-3' and 5'-ATTTCGCTTTCAGCAGTGGT-3'. The RT-PCR products were sequenced to verify the specificity of PCR amplifications.

## Additional data files

The following additional data are available with the online version of this paper. Additional data file 1 contains the putative fold back secondary structure predicted using the MFOLD program. Additional data file 2 contains the predicted targets of conserved and newly identified wheat miRNAs.

## Supplementary Material

Additional data file 1 Putative fold back secondary structure predicted using the MFOLD program. Click here for file

Additional data file 2 Predicted targets of conserved and newly identified wheat miRNAs. Click here for file
