# Supplementary material for: Cloning and characterization of microRNAs from wheat (Triticum aestivum L.)
Source: Genome Biol. 2007 Jun 1;8(6):R96. doi: 10.1186/gb-2007-8-6-r96 (PMC2394755; doi:10.1186/gb-2007-8-6-r96)
Supplement: Additional data file 1 — Putative fold back secondary structure predicted using the MFOLD program. [file gb-2007-8-6-r96-S1.rtf]

Predicted fold-back structures using miRNA containing EST sequences


miR159 : TTGGATTGAAGGGAGCTCTG 

CA731881

 ug        a   u     uga  -    uac  ga    u   u-  a   g    u     guuc    uau   a    auaga 
g  gagcuccu uca uccaa   ag gguc   cg  aggg uug  gc gcu cucg ucaug    ccac   ccu ucucc     \
c  cucgaggg agu agguu   uc ccag   gc  uccc agc  cg cga gagc aguac    ggug   gga agagg     a
 gu        a   u     ug-  g    uc-  --    u   uu  c   g    c     guuu    ucc   g     AGCAA  


CA484819 

   c  u                  aa     u   gc   -    c   ug    g     u    .-g      u    c--        .-guuca  a     cuc 
gga gu gagcuccuuucgguccaa  agggg guu  ugu gggu gau  agcu cuggg caug   aucccg uagc   uacuccau       uc uucag   g
ucu cg cucgagggaaguuagguu  ucucc cag  aca ccca cua  ucga gaccc guac   uagggu guug   augaggua       ag aaguc   a
   a  u                  --     -   gu   u    a   cg    g     c    \ -      u    cuc        \ -----  a     uag

                           

miR160 : TGCCTGGCTCCCTGTATGCCA

CJ641547:

  
    A---------- GA  GA     U  AAACA    GA     C       C         CU    -   CU      UCCCCA 
               G  AG  AGAGG GA     AUGG  UAUGC UGGCUCC UGUAUGCCA  CGCG UAG  GCCAAC      A
               C  UC  UCUCU CU     UGCC  GUACG ACCGAGG GCGUGCGGU  GCGC AUC  CGGUUG      A
    UCUCUCUCUCU UC  UC     -  CUCUC    A-     A       A         AG    C   U-      CGUUCC 


miR164 : TGGAGAAGCAGGGCACGTGCA

CA704421


          c             c  uuucca  u    auuc      uc       .-ccg|     g     uu 
gguggagaag agggcacgugcau ca      gc cggc    ccggcg  cggccgg     gcugcc cggcc  g
ccaccucuuc uuccguguacgua gu      cg gccg    ggccgc  gccggcc     ugaugg gucgg  c
          u             c  ucc---  -    gc--      gu       \ ---^     -     uc 


miR167 : TGAAGCTGCCAGCATGATCTA

CK209908
           
C----------       ----     C      A      A   ------------     UAGCU 
           UGCCCAA    GGGAA GAGUGA GCUGCC GCA            UGAUC     \
           ACGGGUU    CCCUU UUCACU CGACGG CGU            ACUAG     C
UCUUCAUCAAC       GCCG     C      C      -   CGCAAAGAACAA     UGAGC 


CK209889

   CU   CAA     C      A    ACA   ------------     UAUCU 
     GCC   GGGAA GAGUGA GCUG   GCA            UGAUC     \
     CGG   CCCUU UUCACU CGAC   CGU            ACUAG     C
   G-   ---     C      C    GG-   CGCAAAGAACAA     UGAGC 
         


miR169 : CAGCCAAGGATGACTTGCCGA


BJ225371

         
AUG   UGU          A--      G     C       C     CA      CG  --  AC   --        U 
   GGG   GGUAAGAGGU   CUUCGG UAGCC AGGGUGA UUGCC  UGGAAC  CC  GG  GCC  GCUCUCUG \
   CCC   CCAUUCUCCG   GAAGCU AUCGG UCCUACU AACGG  ACUUUG  GG  CC  CGG  CGAGAGAC G
A--   ---          GUA      -     U       A     AC      AG  AA  GU   CU        G 
 

miR171 : TGATTGAGCCGTGCCAATATC

CD910903

U-------    U  U    AU         U  A    C     -    -   .-U   G 
        GGAA GG CACU  GAUGUUGGC CG CUCA UCAGA CCAC GCC   GCC G
        CUUU UC GUGA  CUAUAACCG GC GAGU AGUCU GGUG UGG   CGG C
CGUACGUA    C  -    CU         U  C    U     C    G   \ -   C 
    


miR399 : UGCCAAAGGAGAAUUGCCC

CJ666653

                  .-A     C      C        .-GUG    -      A   AUGAU   G 
 UCGUGUG--UGAAUCAC   GGGCG UUCUCC UUGGCACG     GCAU GCAUGU CAU     GGU G
 AGCGCAC  ACUUGGUG   CCCGU AAGAGG AACCGUGC     CGUG CGUGCG GUG     CCG U
        \         \ -     U      A        \ ---    G      A   GU---   A 
            


miR408:AUGCACUGCCUCUUCCCUGGC

BE419354

 AUUUUGUGAGU          GA     CA---    U  AGCA    A     U      A-  .-AA  A    UAC 
           GGAGAGGGGG  GGAGA     GGGA GG    GAGC AGGGA GAGGCA  GC    CA AAUU   C
           UCUCUCCCCC  CCUCU     CCCU CC    CUCG UCCCU CUCCGU  CG    GU UUAG   A
U----------          UC     CUAAA    C  ----    G     U      CA  \ --  A    UCC 
                                           


miR444 : UUGCUGCCUCAAGCUUGCUGC


CA596074
                  
GACUCGAGA             C    C     A       A         U             G        -  .-ACACC   AUUA    C 
         UAUGCAUGU--GG GGCA CGAGC UGAGGCA CAACUGCAU ACUUGCGGGGAAG CGCAAGUA GG       UGC    CUUG A
         AUAUGUACA  CC CCGU GUUCG ACUCCGU GUUGACGUA UGAACGUUCUUUC GUGUUCAU CC       ACG    GAAC A
ACUAUAUAG         \   U    C     A       C         C             G        A  \ -----   CG--    A 
    


Predicted fold-back structures using new miRNA containing EST sequences


TamiR501: UAGUACCGGUUCGUGGCACGAACC


CA718024 

GUUUGAAAAUGGGA--  -      AC     C                    A  UCUU 
                CC UUUAGU  CGGUU GUGGCACGAACCGGGACUAA GG    \
                GG AAAUCA  GCCAA CACCGUGCUUGGCCUUGAUU CC    C
CGCUGAUUUCGAGGAG  A      GC     A                    A  CCAA
 


TamiR502 : CACUACAUUAUGGAAUGGAGGGA


                  a   a      gc  gcgcu    gag  c-           a          caa       aac           a           aau    aacuucu    a cauacucccuccguucca aau uagugc  cc     uccg   gu  caacuuugacc uaaauuuaac   ugagacc   ugcggcgggag aaaaauuauau   ugaa       uucg a
guaugagggagguaaggu uua aucacg  gg     aggu   cg  guugaaacugg auuuaaauug   guucugg   acgccgccuuc uuuuuaauaua   acuu       aagc u
                  a   c      aa  agau-    gca  aa           c          aug       cua           g           guc    -------    a 


Ta-miR503 : UGGCACGGCGUGAUGCUGAGUCAG


    A-      G    UGA-     .-C     G   GGAGGAGGA         G        GACG   G  -        C 
      GCGUCA CGUG    UGCGC   AGCGC CGC         GCUGGCUGG UCAGCGUC    GCG CG GCCGUGUG U
      UGCAGU GCGC    GCGCG   UCGUG GUG         UGACCGACU AGUCGUAG    UGC GC CGGUGCAU C
    GG      -    UGGG     \ -     -   AGG------         G        ----   G  A        C 


TamiR504 : ACAUUCUUAUAUUAUGAGACGGAG

 
T. aestivum
                   
CCC  C                               A         GU 
  GGG AGGUACUCCCUCCGUCCCAUAAUAUAAGAAC UUUUUGACA  G
  CCC UUUAUGAGGGAGGCAGGGUAUUAUAUUCUUG AAAAACUGU  U
     -                               C         GA 

T. monococcum


  aa       -          c            a   cugg 
    ucccucu ucccauaaua aagaguguuuug aca    \
    agggagg aggguauuau uucuuacaaaac ugu    u
  --       c          a            a   caug


TamiR505 : AGUAGUGAUCUAAACGCUCUUA 


ua    u                       u                 ua 
  uaua guacucccucuguaaagaaauau agaguguuuagaucacu  \
  augu caugagggaggcauuucuuuaua ucucgcaaaucuaguga  a
--    -                       u                 ug 


TamiR507: UCCGUGAGACCUGGUCUCAUAGA


Ta.30511-Ta#S18011750


    CCACCAUG        .-AAAAA    A          A       AUAAAU-             CC      A      G                 G  AU 
            CCAGUGGG       AAUU UAUGAGACCA GUCUCAU       CAGGUGAGACCCG  UUGAUG AUGACA GUGGCAUUCAC--CCCU AU  \
            GGUUACCC       UUAA AUAUUCUGGU CAGAGUG       GUCCACUCUGGGU  AAUUAC UACUGU CACUGUAAGUG  GGGG UG  C
    A-------        \ -----    G          C       CCUAAUC             AA      C      G           \     G  GA 

 

AY616458
 
                  
         A                  AUAAAU-             C       A          G                       A     -      -    A 
AAAAAAAUU UAUGAGACCAGGUCUCAU       CAGGUGAGACCCG CCUGAUG AUGACAUGUG CAUUCACAAAUCACAAAGCAUCU AUCUC UCCCCC CCUG U
UUUUUUUAA AUACUCUGGUCCAGAGUG       GUCCACUCUGGGU GGACUAC UACUGUGCAC GUAAGUGUUUAGUGUUUCGUAGG UGGGG GGGGGG GGAC U
         G                  CCUAAUC             A       C          A                       -     U      U    U 


AY616459

           a                  aua   ---             c       a     a 
ggaaaaaaauu uaugagaccaggucucau   gau   caggugagacccg ccugaug augac c
ccuuuuuuuaa auacucugguccagagug   cua   guccacucugggu ggacuac uacug g
           g                  c--   auc             a       c     u 


TamiR508: GCAGGACGUGAAGAGCGAGUCC
   
AGTACC-  G  TAGCTTC         A--    C   ---           ---------      GGG 
       GA TC       CGGCCTTGG   CTCG TCT   TCACGTCCTGC         GTCGTG   A
       CT AG       GCCGGGACC   GAGC AGG   AGTGTAGGGCG         CAGCGC   T
TAATGTA  G  TTTCCTT         CTC    T   AAG           GTGCGGTAA      AGG 
     


TamiR509 :AACCAACGAGACCAACUGCGGCGG


  auucaug                            aac  c       a     a    auc        auauca 
         uuuggccguaaauuuaaccaacgagacc   ug ggcggga caaaa uuau   gcugaauu      a
         aaacuggcauuuaaauugguuguucugg   ac ccgcccu guuuu gaua   ugacuuaa      a
  -------                            cuu  a       c     g    cga        ncauaa 


TamiR511:  UCCUUCCGUUCGGAAUUAC

  
.-GTCCC     C                TA  ACAA        TAAAA     A   ATATA 
       GCTCC TCTGTTCGAAATTACT  TC    AAATGGAT     TGGAT TAT     \
       TGAGG AGGCAAGCTTTAATGA  AG    TTTACTTA     ATCTA ATA     A
\ -----     A                GC  ATTC        CATGG     C   AAATC 
 

TamiR512 :  UACUACUCCCUCCGUCCGAAA


       a  a     g     ugcuu   uac               a     c              a   a                 acuaa 
auaugag gc ugggu agagg     agu   uacucccuccguccg aaaua uugucaucaaaaug aua aaggggauguaucuaga     \
uguauuu cg guucg ucuuc     ucg   augagggaggcaggc uuuau aacaguaguuuuac uau uuccccuacauagaucu     a
       g  -     g     u----   uua               c     c              c   c                 acaua 


TamiR513: CAGCGAGCCAGCGGAGACCGGCAG

 
TCG     T            T- TG    TCT     T   TC   --   -      -     G     .-CGGA     .-TT     T   T  GG 
   ACGCG CCGG--GCAGCG  C  GTGG   CCGGC GCC  CCA  GGC TTGTCG TCCAT GTGGA      GGCAG    GGGAG GGA CT  C
   TGTGC GGCC  CGTCGC  G  CGCC   GGCCG CGG  GGT  CCG AGCAGC AGGTG TACCT      CCGTT    CCCTT TCT GA  A
---     -    \       TC GT    TCT     T   TT   TC   C      G     G     \ ----     \ --     C   C  GC 
 

TamiR515: UAGUACCGGUUCGUGGCUAACC


  A  C       .-GC          -           -      UUA 
   GG GGGAGGA    UCUUUAGUAC CGGUUCGUGGC GAACCU   G
   CC CCCUUCU    AGAAAUCAUG GCCAAGCACCG CUUGGG   C
  C  -       \ --          N           U      CCA 


CD908217

uc------     -   a   gg                       g    u--    uuua 
        caacg ccu gcc  gccgcucucauuaguaccgguuc uggc   aacc    \
        guugu gga cgg  uggugggaguaaucauggccaag accg   uugg    g
gucugacu     a   -   --                       g    ugc    ccac 


TamiR517: CAUAUACUCCCUCCGUCCGAAA


    AUA               A             C        CA      -            ACUAA 
UAAG   UACUCCCUCCGUCCG AAAUACUUGUCAU AAAAUGGA  AAAAGG GAUGUAUCUAGA     \
AUUC   AUGAGGGAGGCAGGC UUUAUGAACGGUA UUUUACUU  UUUUCU CUACAUAGAUCU     A
ACC               C             C        A-      A            ACAUA 


CJ658560

       a   c  uuu     agaua               a           u     c-   a      ag            uguau 
   auuu cgg gc   ucauu     uacucccuccguccg aaauacuuguc ucaaa  uga uaaaag  gauguaucuaga     \
   uaaa guc cg   aguag     augagggaggcaggc uuuaugaacag aguuu  acu auuuuu  cuacauagaucu     u
       -   u  uuc     -----               c           u     ua   -      cu            ugauu 

TamiR518: CAACAACAACAAGAAGAAGAAGAU
 
CC         T                 T-    .-GCTGA     T  T C   G--      C 
  TCTTCTTGT GTTGTTGTTGT--TTTC  CCAT       TCACG CT C CTG   GGGGGC G
  AGAAGAACG CAACAACAACG  AAAG  GGTA       AGTGT GA G GAC   TTTCCG A
A-         T           \     TT    \ -----     -  - C   GTA      G 
 

TamiR519: CUGCGACAAGUAAUUCCGAACGGA

CJ508389

-----     CG    AU---           A                U    U               A UAA 
     CAGUU  UAGU     CUACUCCCUCC UUCGGAAUUACUUGUC CGGA AUGGAUGUAUCUAGA C   A
     GUCGA  GUCA     GAUGAGGGAGG AAGCCUUAAUGAACAG GUCU UAUCUACAUAGAUCU G   A
AAACA     UU    ACUAU           C                C    U               - CAU 


Ta-miR520: UUGUCGCAGGUAUGGAUGUAUCUA

CJ578485
             C                  U          C    UGUAU 
UACUCCCUCCGUU CGAAUUACUUGUCGCAGG AUGGAUGUAU UAGA     \
AUGAGGGAGGCAA GUUUAAUGAGCAGCGUCU UACCUACAUA AUCU     U
             A                  U          A    UGAUU 


TamiR521: UAGUACAAAGUUGAGUCAUC

     
.-CAC   GACGACG     A    CU                    A          C   G         A 
     GAU       AUUAC UAGC  UGU--ACUCCCUCCGUUCCA AAUGGAUGAC CAA UUUGUACUA A
     CUA       UAGUG AUCG  GCA  UGAGGGAGGCAAGGU UUAUCUACUG GUU AAACAUGAU G
\ ---   AAAGGAA     -    UU   \                C          A   G         U 
     


TamiR522: GCUUAGAUGUGACAUCCUUAAAA
   
DR733919 
             

aucuuguaua   c   c   ga        a    gaca   cu                ga  u   cuc   u-      cuauuuuuuuuauccua 
          ggu ugu uag  cacaucua augu    uaa  augucacaucuaagcu  ug caa   ugu  uguggu                 \
          uca aca auu  guguagau uaca    auu  uacaguguagauucga  au guu   aua  acaucg                 g
uucuag----   a   a   aa        c    aaa-   cc                gg  -   uau   uu      uuauucuuuauuuuuuu 


TamiR523: AGAGUAACAUACACUAGUAACA

BQ903908

    CAC     A       AUA  C         .-A     A    .-UAGA       AA------|    UUC 
--GC   AGUGG GAGUAAC   CA UAGUAACAU   CACAU UCCC      ACUAUAU        CUACC   A
  CG   UCACC CUCAUUG   GU AUCAUUGUA   GUGUA AGGG      UGGUGUG        GAUGG   U
\   A--     -       AA-  U         \ -     C    \ ----       AAUACAAC^    KGA 


 


Wheat new miRNAs conserved in other monocots. EST sequences were used for predicted fold-back structures.


1). TamiR506: UAGAUACAUCCGUAUCUAGA
 
CJ654792.1

auaa    u                                    a     g        u     a     u       a   c  gu 
    guac cccucuguuccuaa--auacucccuccgucccaaaa uuuug cuuagauu gucua auacg auguauc agu au  u
    caug gggagacaaggauu  uaugagggaggcaggguuuu agaac gaaucuaa cagau uaugc uacauag uua ug  u
ac--    u              \                     a     a        u     c     c       a   -  au 


      
Festuca arundanacea: DT715217.1|


UCACAAUAAGCACCA      CA-   A   A     A       C                 GU   G       UCA          -   AUA 
               UUUCAU   AAA AUU CUACU CCUCCGU UAAAAAUAAGUGUCUUA  UUU UCUAGAU   GAUGUAUCUA AAC   U
               AAAGUA   UUU UAA GAUGA GGAGGCA GUUUUUAUUCACAGAGU  AAA AGAUCUA   CUACAUAGAU UUG   U
ACCC-----------      UUG   A   -     G       U                 UG   A       UGC          G   AUU 


2). TamiR510 :  in wheat:  UCCACUAUGGACUACAUACGGAG

              C                A    A   CC            T      GCAAAA  A   AATCTACA 
GTACTCTCTCCGTT CTAAATATAAGTCTTT TAGA ATT  ACTATGGACTAC TACGGA      TA ATG        C
TATGAGGGAGGCAA GATTTATATTCAGAAA ATCT TAA  TGATACTTGGTG ATGCTT      AT TAC        T
              A                C    C   AA            T      ACATAC  C   GTAAAATC 
 


TamiR510 in Barley

acuau         gug     .-cuccuucc     u          ca            c         u         u          ac 
     aguaauuau   augua          guucc aaauauaagu  uuuuagagauuc acuauggac acauacgga gua--uauag  \
     ucauugaua   uguau          caagg uuuauauuca  aaaaucucuaag ugaugccug uguaugccu cgu  auguc  a
gugu-         gua     \ --------     u          ua            a         u         -   \      au 


3). TamiR514: CCUCCGUCUCGUAAUGUAAGACG


UUUUUUUA  CA     .-AA   CUU     -  -                          .-UAUAA   U 
        GU  UUGUA    UGU   AUACU CC CUCCGUCUCAUAAUGUAAGACGUUUU       GCUA \
        CG  AACAU    ACG   UAUGA GG GAGGCAGGGUAUUACAUUCUGCAAAA       UGAU G
CUUGCAN-  NA     \ --   UU-     U  U                          \ -----   U 


In rice:
   
       UC             U     A       CC                                  A   AAU 
AGCUACU  CUCCGUUUCGUAA GUAAG CAUUCUA  AUUUUCUAUAUUUAUAUUAAUGUUAAUGAAUCUA AUA   A
UCGAUGA  GAGGCAAAGUGUU CAUUC GUAAGAU  UAAAAGAUGUAAGUAUAAUUAUAAUUACUUAGAU UAU   U
       GA             U     A       CA                                  C   AUA 
    


4). Ta-miR516 
 

CAUUAUGGAACGGAAGGAG  


rice: AC146937.2 

       c         c                                                      c  cu 
 uacucc uccguuuca aauguaagucauucuagcauuuuccacauucauauagguguuaaugaaucuaga au  a
 augagg aggcaaggu uuacauucaguaagauuguaaaggguguaaguauauccacaauuacuuagaucu ua  u
       a         a                                                      a  ua 

OsmiR468 or TamiR516


aauu|    c         a      cg       a   a                    a  a          ga 
    acucc uccguuucg aaaaaa  aauuuag auu gaugugauacauucuaguac ac aaucuggaca  g
    ugagg aggcaaggu uuuuuu  uuaaauc uga cuacauuguguaagaucaug ug uuagaucugu  a
----^    a         a      ag       a   c                    a  c          au 
