# Supplementary material for: Cloning and characterization of microRNAs from wheat (Triticum aestivum L.)
Source: Genome Biol. 2007 Jun 1;8(6):R96. doi: 10.1186/gb-2007-8-6-r96 (PMC2394755; doi:10.1186/gb-2007-8-6-r96)
Supplement: Additional data file 2 — Predicted targets of conserved and newly identified wheat miRNAs. [file gb-2007-8-6-r96-S2.doc]

Additional file 2 Predicted targets of conserved and newly identified wheat miRNAs

| miRNA family | miRNA/mRNA $ | Predicted target unigene/ EST # | Target gene family | Target site |
| --- | --- | --- | --- | --- |
| TamiR156/157 | CACGAGUGAGAGAAGACAGU 5' |  |  |  |
|  | AUGCUCUCUCUCUUCUGUCA | Ta.3711 (1) | *Squamosa promoter binding protein (SBP)* | ORF |
|  | AUGCUCCCUCUCUUCUGUCA | Ta.6374 (1) |  | 3'-UTR |
|  | GUGCUCUCUCUCUUCUGUCA | Ta.7012 (1) |  | ORF |
| TamiR 159/319 | GUCUCGAGGGAAGUUAGGUUU 5' |  |  |  |
|  | UGGAGCUCCCUUCAUUCCAAU | Ta.38051(2) | *transcription factor GAMYB* | ORF |
|  | UGGAGCUCCCUUCACUCCAAU | Ta. 24098 (2) |  | ORF |
| TamiR 160 | ACCGUAUGUCCCUCGGUCCGU 5' |  |  |  |
|  | AGGCAUACAGGGAGCCAGGCA | Ta.13246 (1) | *auxin response factor* | ORF |
|  | AGGCAUACAGGGAGCCAGGCA | CJ660563 (1) |  | ORF |
|  | AGGCAUACAGGGAGCCAGGCA | CJ728091 (1) |  | ORF |
| TamiR 164 | ACGUGCAUGGGACGAAGAGGU 5' |  |  |  |
|  | AGCAAGUGCCCUGCUUCUCCA | Ta.12537 (3) | *NAC1 transcription factor* | ORF |
|  | CGCACGUGACCUGCUUCUCCA | Ta.12286 (3) |  | ORF |
|  | AGCAAGUGCCCUGCUUCUCCA | Ta.5127 (3) |  | ORF |
|  | CGCACGUGACCUGCUUCUCCA | Ta.33080 (3) |  | ORF |
| TamiR 165/166 | CCCUUACUUCGGACCAGGCU 5' |  |  |  |
|  | UGGGAUGAAGCCUGGUCCGG | Ta.1808 (3) | *class III homeodomain-leucine zipper protein* | ORF |
| TamiR 167 | GUCUAGUACGACCGUCGAAGU 5' |  |  |  |
|  | GAGAUCAGGCUGGCAGCUUGU | Ta.9550 (3) | *auxin response factor 8* | ORF |
|  | UAGAUCAGGCUGGCAGCUUGU | Ta.9398 (3) |  | ORF |
|  | UAGAUCAGGCUGGCAGCUUGU | Ta.6394 (3) |  | ORF |
| TamiR 168 | CAGGGCUAGACGUGGUUCGCU 5' |  |  |  |
|  | UUCCCGAGCUGCACCAAGCGC | Ta.34670 (2) | *Argonaute protein* | ORF |
|  | UUCCCGAGCUGCACCAAGCGC | Ta. 2949 (2) |  | ORF |
| TamiR 169 | AGCCGUUCAGUAGGAACCGAC 5' |  |  |  |
|  | CUGGCAAAUCAUCCUUGGCUU | Ta.10047 (2) | *CCAAT-binding transcription factor* | 3'-UTR |
|  | CAGGCAACUCAUCCUUGGCUU | Ta.27041 (2) |  | 3'-UTR |
|  | CUGGCAAAUCAUCCUUGGCUU | Ta.29451 (2) |  | ORF |
|  | CUGGCAACUCAUCCUUGGCUU | Ta.27574 (2) |  | 3'-UTR |
| TamiR 170/171 | CUAUAACCGUGCCGAGUUAGU 5' |  |  |  |
|  | GAUAUUGGCGCGGCUCAAUCA | Ta.39354 (1) | *scarecrow-like protein* | ORF |
| TamiR 172 | UACGUCGUAGUAGUUCUAAGA 5' |  |  |  |
|  | CUGCAGCAUCAUCAGGAUUCU | CA486144 (2) | *APETALA2 protein AP2* | ORF |
| TamiR 390 | target genes not found |  |  |  |
| TamiR 393 | CUAGUUACGCUAGGGAAACCU 5' |  |  |  |
|  | AGACAAUGCGAUCCCUUUGGA | Ta.23215 (3) | *transport inhibitor response TIR1* | ORF |
| TamiR 396 | target genes not found |  |  |  |
| TamiR 397 | target genes not found |  |  |  |
| TamiR 399 | target genes not found |  |  |  |
| TamiR 408 | CGGUCCCUUCUCCGUCACGUC 5' |  |  |  |
|  | CCCAGGGAAGAGGCAGUGCAG | Ta.1725 (1) | *basic blue copper protein* | ORF |
|  | GCCAGGGCAGAGGCAGUGCAG | Ta.20960 (1) |  | ORF |
|  | CUCAGGGAAGAGGCAGUGCGG | Ta.30891 (3) |  | ORF |
| TamiR 444 | CGUCGUUCGAACUCCGUCGUU 5' |  |  |  |
|  | GCAGCAAGCUUGAGGCAGCAA | Ta.38864 (0) | *MADS-box transcription factor* | ORF |
|  | UCAGCAAGCUUGAGGCAGCAA | Ta.41906 (1) |  | ORF |
| TamiR 479 | target genes not found |  |  |  |
| TamiR 501 | target genes not found |  |  |  |
| TamiR 502 | AGGGAGGUAAGGUAUUACAUCAC 5' |  |  |  |
|  | UCCCUCCAUUCCAUAAUGUAGUG | Ta.14098 (0) | *Unknown protein* | 3'-UTR |
| TamiR 503 | target genes not found |  |  |  |
| TamiR 504 | GAGGCAGAGUAUUAUAUUCUUACA 5’ |  |  |  |
|  | CUCCGUCUCAUAAUAUAAGAAGUU | Ta.5303 (2) | *Unknown protein* | ORF |
|  | CUCCGUUUCAUAAUAUAAGAACGU | Ta.26335 (1) | *aspartic-type endopeptidase/ pepsin A* | 3'-UTR |
| TamiR 505 | AUUCUCGCAAAUCUAGUGAUGA 5' |  |  |  |
|  | UAAGAGCGUUUAGAUCACUACU | Ta.30793 (0) | *Transcription elongation factor 1* | 3'-UTR |
|  | UAAGAGCGUUUAGAUCACUACU | Ta.21208 (0) | *ferric reductase* | 3'-UTR |
|  | UAAGAGCGUUUAGAUCACUACU | Ta.4521 (0) | *Unknown protein* | 3'-UTR |
| TamiR 506 | AGAUCUAUGCCUACAUAGAU 5' |  |  |  |
|  | UCUAGAUACGGAUGUAUCUA | AB182944 (0) | *knox1b homeobox protein* | ORF |
|  | UCUAGAUACGGAUGUAUCUA | Ta.41718 (0) | *Unknown protein* | 5'-UTR |
| TamiR 507 | AGAUACUCUGGUCCAGAGUGC-CU 5' |  |  |  |
|  | UCUAUAAGACCAGGUCUCACGTGA | AY951950(2) | *CRT/DRE binding factor 10* | 3'-UTR |
| TamiR 508 | target genes not found |  |  |  |
| TamiR 509 | GGCGGCGUCAACCAGAGCAACCAA 5' |  |  |  |
|  | CCGCCGCAGUCGGUCUCGUUGGUU | Ta.2441 (1) | *putative UVB-resistance protein* | 3'-UTR |
| TamiR 510 | target genes not found |  |  |  |
| TamiR 511 | target genes not found |  |  |  |
| TamiR 512 | AAAGCCUGCCUCCCUCAUCAU 5' |  |  |  |
|  | UUUCGGACGGAGGGAGUAGUA | Ta.16121 (0) | *ATP binding / ATP-dependent helicase/ DNA binding / helicase/*  *nucleic acid binding / protein binding / ubiquitin-protein*  *ligase/ zinc ion binding* | 3'-UTR |
| TamiR 513 | GACGGCCAGAGGCGACCGAGCGAC 5' |  |  |  |
|  | CUGCCGGUCUCCGCUGGUUCGCUG | Ta.5918 (1) | *Unknown protein* | ORF |
| TamiR 514 | GCAGAAUGUAAUGCUCUGCCUCC 5' |  |  |  |
|  | CGUCUUACAUUAUGGGACGGAGG | Ta.7645 (0) | *Unknown protein* | 3'-UTR |
| TamiR 515 | target genes not found |  |  |  |
| TamiR 516 | target genes not found |  |  |  |
| TamiR 517 | target genes not found |  |  |  |
| TamiR 518 | target genes not found |  |  |  |
| TamiR 519 | AGGCAAGCCUUAAUGAACAGCGUC 5' |  |  |  |
|  | UCCGUUCCAAAUUACUUGUCGCAA | Ta.39646 (3) | *early light-inducible protein ELIP* | 3'-UTR |
| TamiR 520 | target genes not found |  |  |  |
| TamiR 521 | CUACUGAGUUGAAACAUGAU 5' |  |  |  |
|  | GAUGACUCAACUUUGUACUA | Ta.40222 (0) | *expansin like protein A* | 3'-UTR |
| TamiR 522 | AAAAUUCCUACAGUGUAGAUUCG |  |  |  |
|  | UUUUAAGGAUGUCACAUCUAAGC | AF021243 (0) | *translation initiation factor 4B* | 5'-UTR |
| TamiR 523 | target genes not found |  |  |  |

$: the nucleotides underlined represent the mismatches between miRNAs and target mRNAs.

#: the numbers of mismatches between miRNAs and mRNAs are indicated in parentheses.
